# Cost-Effectiveness of Colorectal Cancer Screening in the Dominican Republic

**DOI:** 10.21203/rs.3.rs-7829020/v1

**Published:** 2025-11-19

**Authors:** Alice Agyekum, Sophie Wagner, Karla Marie Disla Pineda, Dolores Mejia de la Cruz, Katherine Crew, Chin Hur, Yoanna Pumpalova

**Affiliations:** Columbia University Irving Medical Center; Columbia University Irving Medical Center; Hospital General de la Plaza de la Salud; Hospital General de la Plaza de la Salud; Columbia University Irving Medical Center; Columbia University Irving Medical Center; Columbia University Irving Medical Center

## Abstract

**Background:**

Colorectal cancer (CRC) incidence is increasing worldwide, particularly in middle-income Caribbean countries like the Dominican Republic (DR), where CRC is now the third-most prevalent cancer. Despite this trend, no national or regional CRC screening guidelines exist across the Caribbean region, and screening is not routinely performed in the DR. In this study, we evaluated the cost-effectiveness of four CRC screening strategies versus no screening.

**Methods:**

We developed a Markov model of CRC to compare no screening (natural history, NH) to four screening strategies: colonoscopy every ten years (Colo), sigmoidoscopy every five years (Sig), biennial fecal immunohistochemical test (FIT), and biennial fecal occult blood test (FOBT), from age 45 to 75. Model inputs were derived from international trials, GLOBOCAN, SEER, and local data. We assumed 60.6% adherence to screening for all strategies. The primary outcome was the incremental cost-effectiveness ratio (ICER) per disability adjusted life year (DALY) averted. Secondary outcomes included CRC incidence, mortality, and number of lifetime colonoscopies.

**Findings:**

In the NH strategy, 2.3% developed and 1.0% died from CRC, with a lifetime cost of $73.89 per person. FIT reduced CRC incidence by 30% and death by 37%, with a lifetime cost of $101.39 and ICER of $2,134.75 per DALY averted. Colo was more effective, reducing CRC incidence by 50% and death by 52%, but exceeded the willingness-to-pay threshold (ICER: $12,903.51). FIT required 371 lifetime colonoscopies per 1,000 people vs. 1,953 for Colo. Results were most sensitive to screening test performance and test costs.

**Interpretation:**

Biennial FIT is a cost-effective CRC screening option for the DR, averting a significant number of CRC cases and deaths at low incremental cost. Inclusion of local data on effectiveness and acceptability of CRC screening modalities would strengthen results. These findings can inform development of CRC screening guidelines in the DR and the wider Caribbean region.

## Introduction

Colorectal cancer (CRC) is the third-most diagnosed cancer and second-leading cause of cancer death worldwide.^1^ In high-income countries (HICs), CRC incidence and mortality have declined due to screening and improved treatments. In contrast, middle-income countries (MICs) are experiencing rising CRC incidence, driven by lifestyle and dietary changes associated with economic development, including increased red meat and processed food consumption, decreased fiber consumption, obesity, decreased physical activity, increased alcohol consumption, and smoking.^1, 2^ Notably, CRC is more often diagnosed at a more advanced stage and in younger persons in MICs, with incidence expected to rise further as these populations age.^2^

This global shift is reflected in Latin America and the Caribbean, where decades of economic growth is projected to result in a 60% increase in CRC incidence by 2030.^3^ The Dominican Republic (DR) is an upper-middle income country that shares the Caribbean Island of Hispañola with Haiti and has a population of approximately 11.8 million.^4^ The DR has had major improvements in health indicators, including infant mortality and life expectancy, but these gains have been coupled with a rise in lifestyle-related cancers.^4, 5^ Between 1990 and 2017, CRC incidence in the DR increased by 87.7%, and although the absolute incidence is low compared to that in HICs (13.9 versus 39.1 per 100,000 person-years respectively in the DR and US in 2017), CRC is already the third-most prevalent cancer in the DR.^6^ Importantly, standard treatments for CRC, including surgery, radiation therapy, and chemotherapy, are available in both the public and private healthcare sectors, although barriers to access exist, including out of pocket costs and availability of diagnostic testing and treatment in rural parts of the country. Despite availability of treatment, CRC case fatality is 60% higher in the DR compared to the US,^3, 7^ a disparity likely driven by late stage at CRC diagnosis, the most important prognostic factor in CRC.

CRC is both preventable and highly treatable when detected early, with an average 5-year overall survival of 91% for localized CRC.^8^ However, the DR lacks national CRC screening guidelines and screening is not routinely performed, leading to late stage at diagnosis.^7^ Single-institution retrospective data from the DR (Rosa Emilia Sánchez Pérez de Tavares National Cancer Institute [INCART] and Hospital General de la Plaza de la Salud [HGPS]) shows that 35–58% of CRCs are diagnosed at stage IV, compared to 23% in the US.^7, 8^ Randomized controlled trials, observational trials, and modeling studies in HICs show that population-level CRC screening is cost effective, results in CRC down-staging, and reduces CRC mortality by 20–50%, depending on screening modality used and screening uptake.^9, 10^ However, few studies have evaluated the acceptability, efficacy and cost-effectiveness of national CRC screening programs in MICs, and many MICs face challenges in implementing early detection and screening programs because of limited infrastructure, awareness, and healthcare prioritization.^11, 12^

To date, no Caribbean nation has established a national CRC screening program.^3, 12^ While the Pan American Health Organization’s “Guide on Cancer Control: Early Detection” recommends CRC screening in Latin America and the Caribbean, it offers limited guidance on optimal screening modality, target population, or how to determine cost-effectiveness in local settings.^3^ Given the increasing burden of CRC in the DR, availability of standard-of-care CRC treatment, preponderance of late-stage diagnoses, and absence of national screening guidelines, there is a need for data to inform public health strategies for CRC control in the DR. To address this gap, we constructed a Markov model to evaluate the cost-effectiveness of four CRC screening strategies compared to no screening in the DR. To our knowledge, this is the first study to formally evaluate the health and economic impact of a national CRC screening program in a Caribbean country. Our model may serve as a broader framework for other countries in the Caribbean region.

## Methods

### Study design

We developed a decision-analytic Markov model to assess the cost-effectiveness of various CRC screening strategies in the DR. We simulated screening from ages 45–75 in an average-risk cohort of 100,000 individuals. The screening strategies evaluated were no screening, or natural history (NH), colonoscopy every 10 years (Colo), sigmoidoscopy every 5 years (Sig), biennial fecal immunochemical test (FIT), and biennial fecal occult blood test (FOBT). The model was constructed using Python 3.12.4 and TreeAge Pro Healthcare 2025 R.1. The study is not considered human participants research and thus institutional review board approval was not required. This study followed Consolidated Health Economic Evaluation Reporting Standards (CHEERS) guidelines and referenced the World Health Organization (WHO) guidelines for cost-effectiveness analysis.^13, 14^

### Model natural history

**Figure 1:**
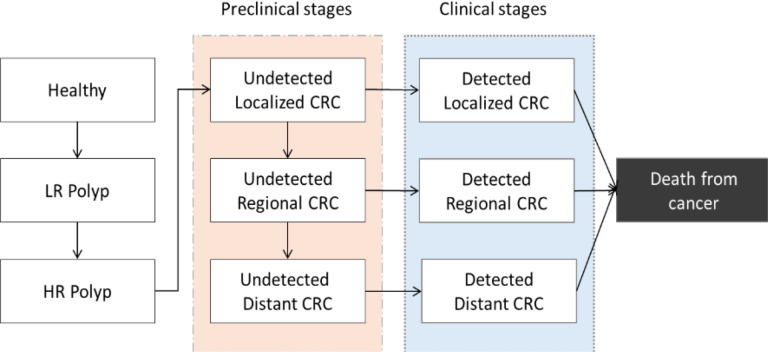
Model schematic for natural history of colorectal cancer. **Note.** *Abbreviations:* LR (low-risk), HR (high-risk) CRC (colorectal cancer).

The model’s natural history component simulates the cohort’s potential progression from a healthy state to CRC in the absence of screening, accounting for changes in risk as they develop adenomas which may advance to invasive cancer over time. Patients enter the model without adenomas at age 20 and are followed until age 100 or death. Over time, patients may develop adenomas, which may progress from low-risk (<10mm) to high-risk (≥10mm), and to preclinical CRC.^15^ Preclinical CRC may progress in stage from local to regional to distant CRC, with an increasing likelihood of detection due to symptoms with increasing stage. Survival outcomes following cancer diagnosis depend on a patient’s age and cancer stage at diagnosis. All-cause mortality rates were derived from WHO lifetables for the DR, and stage-specific cancer-mortality rates were derived from Surveillance, Epidemiology and End-Results (SEER) survival estimates (**Supplementary Table 1**). Once in a detected cancer state, patients are subject to cancer-specific mortality risks for up to 10 years; after this period, only all-cause mortality rates are applied. Individuals may die from causes other than CRC at any age (Figure 1).

Following methodology used in CRC models developed by Cancer Intervention and Surveillance Modeling Network (CISNET), we modeled only the development of cancerous polyps (adenomas); non-cancerous polyps were not included in the model.^16^ We also assumed that each adenoma detected during screening is completely removed, preventing its potential progression to CRC.

#### Natural history calibration

The natural history model was calibrated to CRC incidence and adenoma prevalence targets. CRC incidence was calibrated to SEER data from 1975–1990 to reflect a period with minimal CRC screening in the US.^17^ Adenoma prevalence was calibrated to estimates from published autopsy studies.^18^ We used stepwise simulated annealing to calibrate age-based monthly transition probabilities for adenoma development, progression to preclinical CRC, preclinical CRC progression, and symptomatic detection. Calibration was validated qualitatively and empirically with SEER CRC incidence rates (**Supplementary Figure 1**).

After calibrating the model using US data, we fine-tuned the model using all available local data on CRC incidence and stage distribution in the Dominican Republic. Calibration targets included CRC incidence estimates from GLOBOCAN, a component of the Global Cancer Observatory (GCO) International Agency for Research on Cancer (IARC),^1^ and CRC stage distribution data from the national cancer referral hospital, INCART, in Santo Domingo, Dominican Republic (2017, unpublished data) (**Supplementary Table 2).**

### Screening strategies

Following the calibration of the natural history model, we integrated Colo, FIT, FOBT, and Sig screening strategies. Individuals in the cohort underwent screening beginning at age 45 and continued at the specified intervals until age 75. In the non-colonoscopy strategies, we assumed an individual with a positive test received a follow-up colonoscopy to confirm findings. If a high-risk adenoma was detected on colonoscopy, an individual entered a high-risk adenoma surveillance program, adhering to the European schedule for CRC surveillance until age 85.^19^ Irrespective of the individual’s adenoma or cancer status, if they received a colonoscopy that came back negative (potentially false negative), they did not receive another screening test for 10 years. In the base case, we assumed 60.6% adherence to first-line screening, 100% adherence to follow-up colonoscopy, and 100% adherence to high-risk adenoma surveillance.

### Test performance characteristics

The model’s screening strategies allowed for adenomas or preclinical cancer to be detected based on the sensitivity of the screening test for that condition and, for endoscopic tests, the depth of endoscope insertion. Sensitivities and specificities for each test (colonoscopy, FIT, FOBT, and sigmoidoscopy), as well as probability of complications from endoscopy, were determined from published literature (**Supplementary Table 2**).

### Costs

Costs were calculated from a healthcare payer perspective and included costs of screening tests, complications, and cancer treatment. Costs were derived from the DR’s Seguro Nacional de Salud (SeNaSa) database and HGPS (**Supplementary Table 2**). Costs were converted from Dominican pesos to 2024 US dollars using the Dominican Peso (DOP) to US Dollar (USD) exchange rate of 0.017, based on historical exchange rates, and adjusted for inflation using the Consumer Price Index for health care (**Supplementary Table 2**). Cancer treatment costs included surgery, hospitalization, CT scans, consultations, and chemotherapy. Individuals diagnosed with CRC received treatment specific to their cancer stage at diagnosis.

### Effectiveness

Following common practice in global health research and WHO guidelines for cost-effective analyses, we estimated the effectiveness of each screening intervention by calculating the disability-adjust life years (DALYs) averted for each scenario. Disability weights were applied when undergoing endoscopy, experiencing endoscopy-related complications, undergoing chemotherapy or surgery, and living with cancer. Disability weights for living with cancer were calculated by subtracting health-state utility values for CRC for each stage, obtained from published literature, from 1.^20,21^ Stage-specific disability weights were then applied for 10 years following CRC diagnosis to calculate DALYs over a 10-year period following CRC diagnosis. Costs and DALYs were discounted at a monthly rate of 3%.

### Outcomes

The primary endpoints were DALYs, total costs in 2024 US Dollars, and incremental cost-effectiveness ratio (ICER), calculated as incremental cost in USD per DALY averted. We adopted the WHO definition of cost-effectiveness, defining a willingness-to-pay (WTP) threshold equal to the DR 2024 Gross Domestic Product (GDP) per capita.^14, 22^ A strategy was considered cost-effective if its associated ICER was below a WTP threshold of $11,692/DALY averted. Secondary endpoints included CRC incidence, CRC-specific death, total costs, life expectancy, and number of lifetime colonoscopies per 1,000 individuals screened.

### Sensitivity analyses

We performed deterministic and probabilistic sensitivity analyses to explore uncertainty in model parameters, including costs, test performance, and disability weights. One-way deterministic sensitivity analysis varied one parameter at a time while holding others constant to determine individual effects of parameter uncertainties on model robustness. In a probabilistic sensitivity analysis (PSA), all key parameters are sampled simultaneously from predefined probability distributions. Costs were sampled from Gamma distributions, while probabilities and disability weights were sampled from Beta distributions. The ranges and sampling distributions were determined from literature and common practices in economic evaluations.^23–25^

We additionally evaluated assumptions of screening adherence. We compared model outputs under our base adherence (60% to all initial screening and 100% adherence to follow-up diagnostic tests) to a scenario assuming 100% adherence to all initial screening and follow-up diagnostic tests. Among the two top-performing strategies (Colo and FIT), we conducted further analysis to explore variations in both initial screening adherence and adherence to diagnostic follow-up.

We also assessed model outcomes under different screening and surveillance windows. We modeled alternative start (40 or 50 versus 45 years) and stopping ages (75 versus 85 years) for screening and high-risk adenoma surveillance, respectively. We further explored a scenario of CRC screening using FIT starting at age 45 and switching to Colo at age 50.

Lastly, we performed additional sensitivity analyses to assess how model results changed under different CRC incidence and stage distribution assumptions. We modeled three CRC incidence scenarios: base case, half the base incidence (1/2x), and double the base incidence (2x). We also modeled an alternative stage distribution, using data derived from SEER 8 (1975 – 1985) in place of the stage distribution derived from INCART data. SEER 8 was chosen because this data was collected before widespread screening was practiced in the US, but still reflects an earlier stage distribution compared to the single-institution retrospective data used for our base-case scenario (INCART in Santo Domingo, DR).

## Results

### Base case results

**Figure 2:**
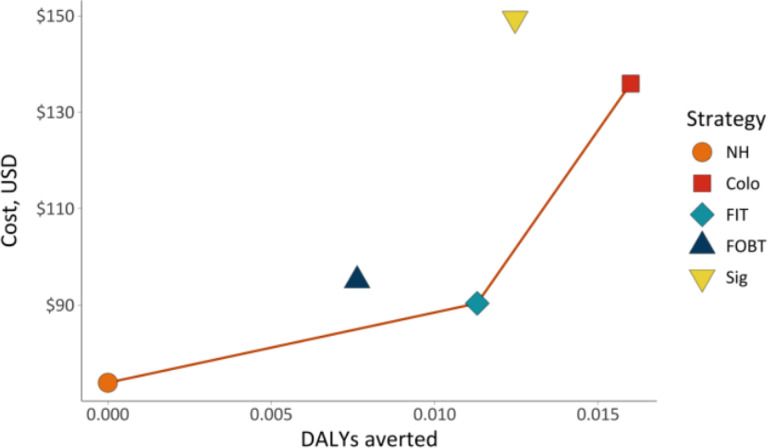
Cost-effectiveness plane of colorectal cancer screening strategies in the Dominican Republic. **Note.** *Abbreviations:* CRC (colorectal cancer), DALY (disability-adjusted life-year), NH (natural history), FIT (biennial fecal immunochemical test), FOBT (biennial fecal occult blood test); Colo (colonoscopy every 10 years), Sig (sigmoidoscopy every 5 years). All costs and DALYs represent per-person estimates accumulated over the modeled time horizon. Strategies connected by the line (NH, FIT, and Colo) lie on the cost-effectiveness frontier, representing the most efficient options. Sig and FOBT are absolutely dominated, meaning they result in higher costs and fewer DALYs averted compared to other strategies.

Model results for CRC screening strategies, including per-person costs, DALYs, CRC incidence and mortality, and number of colonoscopies over the model lifetime are shown in Table 1 and the cost-effectiveness plane in Figure 2. In the no intervention (NH) strategy, 2.3% of the population developed CRC and 1.0% died from the disease. The lifetime cost per person was $73.89, and the average life expectancy was 75.35 years. In the NH arm, 23 lifetime colonoscopies per 1,000 individuals were performed due to symptomatic CRC presentation.

The FIT strategy was cost-effective, with an ICER of $2,134.74 per DALY averted. It increased average life expectancy to 75.37 years, reduced CRC incidence by 30%, and lowered CRC-related mortality by 37% compared to NH. FIT was associated with a mean cost of $101.39 per person and 371 lifetime colonoscopies per 1,000 individuals screened.

The Colo strategy yielded the highest average life expectancy at 75.42 years and achieved the greatest reductions in CRC incidence (50%) and mortality (52%). However, with an ICER of $12,903.51 per DALY averted, Colo exceeded the WTP of $11,692 and was therefore not considered cost-effective compared to FIT. Additionally, the Colo arm required 1,953 lifetime colonoscopies per 1,000 individuals screened.

The FOBT and Sig strategies also reduced cancer burden (CRC incidence and mortality reductions of 21% and 27% and 40% and 42%, respectively, compared to NH). However, both FOBT and Sig strategies were dominated by other strategies, meaning they were more costly and less effective compared to FIT and Colo.

### Sensitivity analysis results

**Figure 3:**
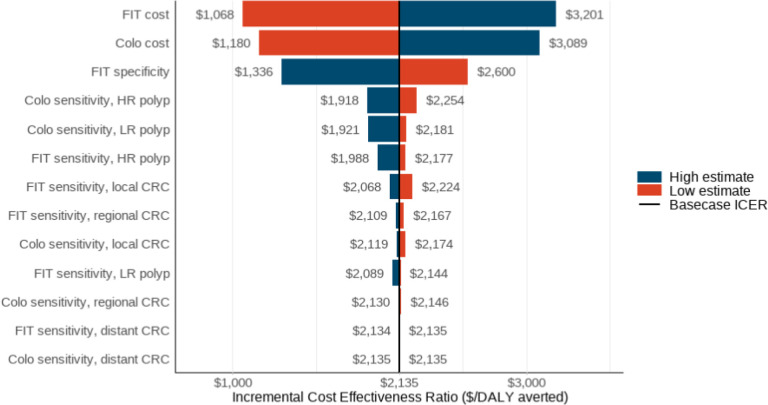
One-way sensitivity analysis comparing biennial fecal immunochemical test (FIT) colorectal cancer screening strategy vs natural history (NH). **Note.** *Abbreviations:* CRC (colorectal cancer), DALY (disability-adjusted life-year), ICER (incremental cost-effectiveness ratio), EV (expected value), NH (natural history), FIT (biennial fecal immunochemical test), Colo (colonoscopy), LR (low-risk), HR (high-risk). Model parameters were independently varied across a set range while all other parameters were held constant at their base-case value. Model parameters for test performance characteristics and costs are shown; parameters with the largest effect on the ICER are shown at the top and those with smallest effect are shown at the bottom. High and low estimate indication on either side of the EV indicate the direction that an increase or decrease in the model parameter changes the ICER. The tornado diagram shown compares no screening with our next most optimal strategy, biennial FIT.

In one-way sensitivity analyses comparing each screening strategy to NH, FIT consistently remained below the WTP threshold, demonstrating robustness to variations in parameter estimates. The model was most sensitive to the cost of FIT, cost of colonoscopy, and the specificity of FIT when comparing NH with FIT (Figure 3).

**Figure 4:**
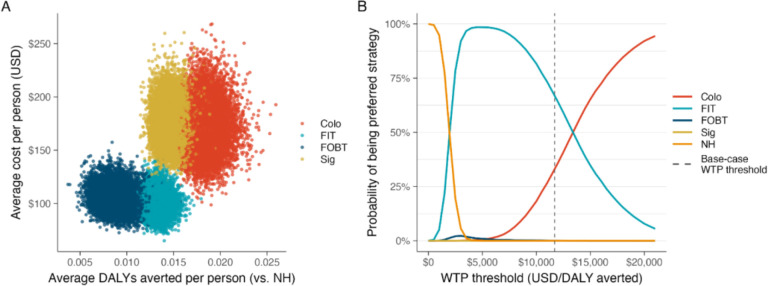
Probabilistic sensitivity analysis results for CRC screening strategies in the Dominican Republic. (A) Costs versus DALYs averted compared to no screening (NH). (B) Cost-effectiveness acceptability curve. **Note.** *Abbreviations:* CRC (colorectal cancer), DALY (disability-adjusted life-year), NH (natural history), USD (U.S. Dollars), WTP (willingness-topay), FIT (biennial fecal immunochemical test), FOBT (biennial fecal occult blood test), Colo (colonoscopy every 10 years), Sig (sigmoidoscopy every 5 years). Probabilistic sensitivity analysis (PSA) was performed for 10,000 simulations, jointly sampling input parameters from predefined distributions. Figure 4A plots the average per-person cost and DALYs averted for each simulation relative to no screening (NH). Points in the bottom left represent simulations with lower costs and fewer DALYs averted, while points in the top right represent higher costs and DALYs averted. Figure 4B presents the cost-effectiveness acceptability curve, which shows the probability that each strategy is the most cost-effective option across a range of WTP thresholds. The dashed vertical line indicates the base-case WTP threshold of $11,694, at which FIT was the most cost-effective strategy in 66.9% of simulations.

Probabilistic sensitivity analysis indicated that with a WTP threshold of $11,692 per DALY averted, FIT was the cost-effective strategy in 66.9% of 10,000 iterations (Figure 4).

We explored how varying levels of screening adherence impacted CRC outcomes and model results. At a 100% adherence rate to initial screening tests and all follow-up diagnostic testing (when applicable), FIT remained the preferred strategy, reducing CRC incidence and mortality by 49% and 61% respectively, with a lifetime cost per person of $119.26 and an ICER of $2,134.75 (**Supplementary Table 3**).

We further compared the performance of FIT and Colo under different follow-up adherence assumptions (**Supplementary Figure 2**). As expected, the Colo strategy consistently produced greater reductions in CRC incidence and mortality at equivalent levels of initial screening adherence. However, similar outcomes were predicted with FIT under more favorable screening adherence levels. For example, a 20% reduction in CRC incidence could be attained with approximately 25% adherence to Colo, 40% adherence to FIT and 100% colonoscopy follow-up, or 55% adherence to FIT and 60% colonoscopy follow-up.

When varying screening start age, FIT was optimal if screening started at age 40 and 45, but Colo became the preferred strategy when screening started at age 50 (**Supplementary Table 4**). These results prompted us to evaluate a scenario of starting CRC screening with FIT at age 45 and switching to Colo at age 50, but this strategy was not on the efficiency frontier compared to the base case screening strategies (**Supplementary Figure 3**). When high-risk adenoma surveillance ended at age 75 (instead of 85 in the base-case), FIT remained the preferred strategy (**Supplementary Table 5**).

Changes in CRC incidence also influenced the optimal strategy. When CRC incidence was halved, FIT remained optimal while when incidence was doubled, Colo emerged as the optimal strategy (**Supplementary Table 6**). We also assessed an alternative CRC stage distribution derived from US SEER 8 data which had a more favorable stage distribution than the single-institution retrospective data used in our base case scenario. Under this assumption, FIT remained the optimal screening strategy (**Supplementary Table 7**).

## Discussion

In this study, we constructed a Markov decision-analytic model to analyze the cost-effectiveness of four established CRC screening strategies in the DR compared to the current status quo of no CRC screening. To our knowledge, this is the first published economic analysis to evaluate the cost-effectiveness of CRC screening in the DR or any Caribbean country. Biennial FIT was the optimal strategy, reducing CRC incidence and mortality by 30% and 37%, respectively, compared to no screening, assuming 60.6% adherence to FIT and 100% adherence to follow-up colonoscopy if FIT is positive. This strategy was estimated to require 371 lifetime colonoscopies per 1,000 persons screened at a lifetime cost of $101.39 per person. The resulting ICER was $2,134.75 per DALY gained, well below our WTP threshold set at the DR’s 2024 GDP per capita of $11,692, and far less than half of this value – and thus considered “very cost-effective” per WHO standards.^26^

Probabilistic sensitivity analysis showed that biennial FIT was the cost-effective strategy in 66.9% of 10,000 iterations of the base-case scenario. However, there were specific scenarios in which colonoscopy became the preferred strategy, namely if CRC screening was initiated at age 50 instead of age 45, and if CRC incidence in the DR doubled. In these scenarios, the ICER for colonoscopy was $9,390.74 and $6,107.10 respectively, which is below our WTP threshold set at the DR’s 2024 GDP per capita, but remains above the WHO’s “very cost-effective” threshold of half the GDP per capita. In a 2023 publication, Pichon-Riviere *et al*. empirically derive country-specific thresholds for health interventions for 174 countries using local health expenditures per capita and life expectancy at birth and show that in upper-middle income countries like the DR, the derived cost-effectiveness threshold per quality adjusted life year (QALY) gained was less than 1x GDP per capita for 100% of the countries included, and less than half the GDP per capita for 76% of countries included.^26^ Based on this analysis, any CRC screening intervention intended to be implemented in the public healthcare sector in the DR should cost less than half the GDP per capita threshold to be considered cost-effective. Only biennial FIT meets this criterion in our analysis. Results from our initial scenario analyses raised the possibility of implementing biennial FIT-based CRC screening for average-risk individuals between the ages of 45–50 and switching to colonoscopy-based CRC screening after age 50, especially if CRC incidence continues to steadily increase in the DR. However, compared to the base case screening strategies, this switching strategy was not on the efficiency frontier in a sensitivity analysis (**Supplementary Figure 3**).

Our results strongly support the cost-effectiveness of CRC screening in the DR, but the practical and logistical steps necessary to implement such a program nationwide are numerous, and difficult to assess using modeling studies alone. Multiple studies, including several meta-analyses, have described the current landscape of CRC screening in low- and middle-income countries, consistently showing that screening in these settings is typically either unavailable or opportunistic in delivery and that most CRC cases globally are diagnosed when patients become symptomatic, as is currently the case in the DR. In MICs that do have CRC screening programs, FIT-based programs are the most common.^11^ Specifically, a systematic review and meta-analysis of CRC screening programs in Latin America identified CRC screening programs in only seven upper-middle or high-income countries in Latin America, with no programs in lower-middle income countries and no programs in Caribbean countries.^12^ Of the 17 identified programs, 13 were FIT-based, while only 4 were colonoscopy-based without prior FIT, likely reflecting the higher cost and resources needed for colonoscopy-based programs. The overall pooled adherence rate to FIT in this study was 85.8% and the follow up colonoscopy rate was 79.6%. These real-world data lend credibility to our base model inputs, which included 60.6% FIT adherence rate and 100% follow-up colonoscopy adherence rate. However studies from Uruguay and Cuba report much lower adherence to FIT (<20% uptake in both countries),^27, 28^ and local data from a pilot CRC screening program in a private sector hospital in Santo Domingo, DR (Hospital General de la Plaza de la Salud) also suggest low adherence to screening FIT (<20%) and low follow-up colonoscopy completion rates of 22% (Pumpalova, unpublished data). To address this limitation, we performed a sensitivity analysis comparing the impact of CRC screening at various adherence rates of the two top-performing strategies (Colo and FIT). Our results show that a 20% reduction in CRC incidence could be achieved with any of the following: 25% adherence to Colo; 40% adherence to FIT and 100% adherence to follow-up colonoscopy; 55% adherence to FIT with 60% follow-up; or 70% adherence to FIT with 40% follow-up. This may be particularly relevant, given evidence that FIT typically has higher uptake than Colo, and emphasizes the importance of patient navigation to ensure adherence to follow-up colonoscopy.^29^

While ours is the first study to evaluate the cost-effectiveness of CRC screening in the DR or any country in the Caribbean region, our findings are consistent with similar analyses done in other middle and upper-middle income countries. In 2012, Pinzon Florez *et al* published a Markov model evaluating the health and economic impact of six CRC screening strategies in Colombia and in 2015 a similar analysis was published for Argentina.^30, 31^ Both models recommended stool-based testing based on costs and cost-effectiveness thresholds, although colonoscopy-based screening strategies were more effective. In October 2024, Lu *et al* published a microsimulation model using Chinese epidemiological data to evaluate the clinical and economic impact of four CRC screening strategies at various national adherence rates.^32^ Like our study, the authors found that all strategies reduce CRC incidence and mortality compared to no screening, with colonoscopy outperforming FIT-based strategies at the same invitation rates. However, CRC screening using colonoscopy was associated with higher costs and a greater number of lifetime colonoscopies and like us, the authors conclude that biennial FIT-based screening strategies are the preferred strategy for China.

In MICs, colonoscopy capacity is often limited by lack of trained personnel, including gastroenterologists, anesthetists, and pathologists, as well as lack of infrastructure such as endoscopy suites. These limitations mean that endoscopy-based one-step CRC screening is unrealistic for much of the world. Our model outputs therefore account not only for cost-effectiveness, but also for feasibility in terms of lifetime colonoscopy demand. In the DR, the Sociedad Domincana de Gastroenterologia has 539 active members, roughly equating to 4.5 practicing gastroenterologists per 100,000 people in the general population, or 4.5 practicing gastroenterologists per 25,640 people within the CRC screening age.^33^ In our model, biennial FIT would require 371 lifetime colonoscopies per 1,000 people screened, equating to 9,275 colonoscopies per 25,640 people screened, or 2,113 lifetime screening colonoscopies per endoscopist, which is well within average expectations for gastroenterologists. By contrast, colonoscopy every 10 years would require 1,953 lifetime colonoscopies per 1,000 people screened, resulting in 11,127 lifetime screening colonoscopies per endoscopist. Additionally, these crude workforce estimates likely overstate true capacity, as both gastroenterologists and endoscopy infrastructure are disproportionately concentrated in larger cities such as the capital, Santo Domingo, leaving much of the country with severely limited access. Implementing a colonoscopy-based strategy will therefore require not only an increase in the number of trained specialists and endoscopy suites, but also a more equitable distribution of services, both of which will take time to achieve. Our model predicts that CRC screening using colonoscopy becomes increasingly cost-effective as CRC incidence increases in the DR, highlighting the importance of beginning investments in endoscopy capacity now to prepare the healthcare system for the growing demand as CRC incidence increases.

Our study has several strengths and some key limitations. We used a validated modeling framework for cost-effectiveness analyses, consistent with prior approaches, and compared multiple guideline-supported CRC screening strategies. We included lifetime colonoscopy demand as a model output, providing insight into feasibility and resource utilization alongside cost-effectiveness. Although this does not fully capture the real-world costs of implementing CRC screening programs in MICs, where limitations in trained personnel, infrastructure, and equipment may pose significant challenges, it is a meaningful starting point for assessing feasibility. We also tested the robustness of our base-case results using a wide range of scenario and sensitivity analyses, which consistently supported biennial FIT as the preferred strategy in the DR. We derived costs using local data from SeNaSa and HGPS; however, this approach does not capture out of pocket costs, as well as differences in costs between public and private sectors, which may limit generalizability. Nonetheless, extensive sensitivity analyses demonstrated that the ICER for biennial FIT remained below the GDP-derived WTP threshold in both one-way and probabilistic sensitivity analyses. Our model incorporated all available data on CRC incidence and stage distribution from the DR, but this data was not always nationally-representative. Specifically, stage-distribution data was derived from just one institution - INCART, the national referral hospital for cancer care in the Dominican Republic. We were unable to obtain nationally representative or single-institution data on CRC mortality from the DR, thus our model used CRC mortality data from SEER 1975–1985 to reflect worse cancer-specific outcomes in MICs. To account for uncertainty in CRC incidence, stage distribution, and outcomes after diagnosis, we calibrated multiple natural history models reflecting a range of plausible assumptions. Despite these strategies, the limited availability of DR-specific data may affect the generalizability of our results and highlights the need for improved cancer data collection in the DR.

Data from high-income regions of the world that have implemented population-level CRC screening using FIT show that at least 50% adherence to initial testing is necessary to meaningfully decrease CRC incidence and mortality on a population level, and that this is best achieved with organized national CRC screening programs.^9^ These studies, local data from DR, and our model all underscore the need for further investigation in the DR to understand what the barriers and facilitators are to improve adherence to CRC screening using FIT, and what interventions are necessary to ensure acceptability of FIT. Such studies are needed before large-scale roll out of CRC screening using FIT should be implemented.

## Conclusion

Biennial FIT is a cost-effective CRC screening option for the DR, and if implemented nationwide would significantly decrease the number of CRC cases and deaths, at an incremental cost that is well below the willingness to pay threshold. Follow-up studies on the effectiveness and acceptability of CRC screening using FIT are needed before CRC screening can be successfully implemented in the DR. Our model can be adapted using local data from other Caribbean countries to inform the development of CRC screening guidelines in this region.

## Supplementary Material

Supplementary Files

This is a list of supplementary files associated with this preprint. Click to download.

- YSPBMCSupplementaryMaterials.docx

## Figures and Tables

**Table 1.** Per-person costs, DALYs, CRC cases, and number of colonoscopies over the model lifetime.

						CRC cases		CRC deaths		
Strategy	Lifetime cost per person (USD)	Incremental cost (USD)	Avg. life expectancy (years)	DALYs averted	ICER/DALY averted	N per 100k	% averted compared to NH	N per 100k	% averted compared to NH	Lifetime colonoscopies per 1000
**NH**	73.89	-	75.35	-	-	2333	-	997	-	23
**FIT**	**101.39**	**27.50**	**75.37**	**0.0129**	**2134.74**	**1636**	**30%**	**627**	**37%**	**371**
**FOBT**	106.46	5.07	75.35	−0.0037	Dominated	1849	21%	728	27%	579
**Colo**	171.43	70.04	75.42	0.0054	12903.51	1170	50%	477	52%	1953
**Sig**	177.90	6.47	75.37	−0.0041	Dominated	1408	40%	579	42%	307

**Note.** *Abbreviations:* CRC (colorectal cancer), DALY (disability-adjusted life-year), Avg. (Average), ICER (incremental cost-effectiveness ratio), NH (natural history), FIT (biennial fecal immunochemical test), FOBT (biennial fecal occult blood test); Colo (colonoscopy every 10 years), Sig (sigmoidoscopy every 5 years).

## Data Availability

The model natural history is made available at https://github.com/CUMC-HIRE/dr-crc-calibration.
